# Growth Factors Assessed during Kasai Procedure in Liver and Serum Are Not Predictive for the Postoperative Liver Deterioration in Infants with Biliary Atresia

**DOI:** 10.3390/jcm10091978

**Published:** 2021-05-05

**Authors:** Omid Madadi-Sanjani, Stephanie Froemmel, Christine S. Falk, Gertrud Vieten, Claus Petersen, Joachim F. Kuebler, Christian Klemann

**Affiliations:** 1 Department of Pediatric Surgery, Hannover Medical School, Carl-Neuberg-Straße 1, 30625 Hannover, Germany; madadi-sanjani.omid@mh-hannover.de (O.M.-S.); froemmel.stephanie@mh-hannover.de (S.F.); vieten.gertrud@mh-hannover.de (G.V.); petersen.claus@mh-hannover.de (C.P.); 2 Institute of Transplant Immunology, Hannover Medical School, Carl-Neuberg-Straße 1, 30625 Hannover, Germany; falk.christine@mh-hannover.de; 3 German Center for Infection Research DZIF, Thematical Translation Unit-Immunocompromized Host (TTU-IICH), 38124 Braunschweig, Germany; 4 Department of Pediatric Pulmonology, Allergology and Neonatology, Hannover Medical School, Carl-Neuberg-Straße 1, 30625 Hannover, Germany

**Keywords:** biliary atresia, BA, liver, serum, growth factors, multiplex, Kasai procedure, liver cirrhosis, liver transplantation, LTX, long term outcome, prognosis

## Abstract

Background: Biliary atresia (BA) is a neonatal cholangiopathy characterized by progressive destruction of the biliary system resulting in liver cirrhosis. Residual bile drainage can temporarily be achieved through Kasai portoenterostomy (KPE) and some children show long-term survival with their native liver. However, most children eventually require liver transplantation (LTX). As several growth factors (GF) and chemokines have been shown to promote fibrogenesis in the liver, we assessed whether GF are predictive for the course of disease. Material and Methods: Liver and sera samples were collected from 49 infants with BA during KPE. Levels of 13 different GF were measured by multiplex immunoassay. Patient outcomes were stratified into favorable (bilirubin < 20 µmol/L at 2-year follow-up) and unfavorable (LTX). GF levels were compared between groups by a *t*-test, correlation coefficients were calculated, and principal component analyses performed. Results: Twenty-two patients showed a favorable and 27 an unfavorable disease course. No relation of GF and outcome could be established. In both groups, high levels of SDF-1alpha/CXCL12 (1473.0 ± 497.5 pg/mL), FGF2 (301.2 ± 207.8 pg/mL), and VEGF-a (209.0 ± 146.4 pg/mL) levels were measured within the liver, followed (in descending order) by PDGF-bb, LIF, GM-CSF, BDNF, VEGF-d, beta-NGF, IL-7, SCF, PIGF-1, and EGF. Serum marker levels showed much higher mean variation compared to hepatic values and no correlation to the protein microenvironment in the liver. Conclusions: Our study demonstrates high amounts of GF in livers from infants with BA at KPE, but no correlation to the outcome or serum values could be established. Our data suggest that local or systemic GF levels are unsuitable for prediction of the disease course. Collectively, we conclude that in BA the degree of proliferative activity caused by GF is a dismissible factor for the further course of disease.

## 1. Introduction

Biliary atresia (BA) is a rare fibro-obliterative disease, manifesting within the first weeks of life, and resulting in end-stage liver cirrhosis [1]. This continuous progress to liver deterioration can be interrupted with the Kasai portoenterostomy (KPE) by restoring some bile drainage in up to 60% of operated infants [2]. However, in the majority of patients, inflammation and fibrosis continue postoperatively, necessitating liver transplantation (LTX), making BA the most common indication for pediatric LTX [3,4]. Advances in diagnostic and therapeutic algorithms have significantly increased the overall survival in BA over the last decades; nevertheless, only 20–50% of children show long-term survival with their native liver [4,5]. Despite intense research, only a few markers are indicative for a favorable post-KPE course of disease, including the age at operation with significantly better results when children are less than 60 days of age at KPE [6]. The most important postoperative marker for the KPE outcome is the serum bilirubin, which shows a significant correlation with the infant’s survival with native liver [7,8]. However, the dynamics of the postoperative liver deterioration are still unpredictable. Observations of histological features in BA show ductular reaction with bile duct proliferation, liver fibrosis in the portal area, bile plugs, and ductal plate malformation in the majority of infants, but these are unpredictive for the further course of disease [9,10,11,12]. Histopathological semiquantitative scoring systems at the time of KPE remained ineffective as predictors of the further course of the disease as well [11,13,14]. Furthermore, local and systemic inflammatory mediators were repeatedly suggested to describe the disease dynamics; however, none of these parameters were proven to have a predictive value [11]. An early prognostic indicator for the post-KPE disease dynamics is crucial to solve the major problem of late referral of patients to pediatric liver units, as registry data show that the pediatric mortality on transplant waiting lists is up to 20% [15].

Various growth factors (GF) have been proven to be elevated during fibrotic processes in cholestatic hepatic disorders [16]. This was attributed to the crucial role of GF in the regulation of liver stem cell differentiation [16]. The balance of the GF-induced stem cell differentiation into hepatocytes, cholangiocytes, and other cell types regulates the development of liver cirrhosis and could, therefore, potentially predict the fibrosis dynamics [17].

Based on this, we hypothesized that the levels of growth factors in liver or sera of infants with BA might be predictive of the course of disease, identify BA patients at risk for a rapid deterioration of liver function and consecutive need of LTX, and thus enabling the early postoperative referral to LTX units. Therefore, we investigated the systemic and local growth factor milieu in a cohort of infants with biliary atresia at the time-point of KPE and correlated the results with the patients’ follow-up data.

## 2. Material and Methods

### 2.1. Ethics

The study protocol is in accordance to the declaration of Helsinki and was approved by the ethic committee of the Hannover Medical School. Written informed consent was obtained from each patients’ parents.

### 2.2. Patients and Follow-Up

Forty-nine infants diagnosed with isolated biliary atresia at the Hannover Medical School between 2004 and 2014 were included in the study. Isolated biliary atresia was distinguished from BA with associated malformations, including splenic malformations (polysplenia, double spleen, asplenia), disorders of visceral symmetry (malrotation, situs inversus), intraabdominal vascular abnormalities (absent inferior vena cava, preduodenal portal vein), and cardiac anomalies [1]. Patients with BA associated with other congenital malformations were excluded from the study.

KPE in all patients was performed by two experienced surgeons. Adjuvant therapies included fat-soluble vitamins, ursodeoxycholic acid, and prophylactic antibiotics. Furthermore, all patients received postoperative budesonide (2 mg per day) through rectal application, starting the fourth postoperative day and continuing for three months following KPE. More than two years of follow-up data were available for all patients. Outcomes were stratified into favorable (jaundice-free (e.g., bilirubin levels < 20 µmol/L) survival with native liver >2 years (JF-SNL)) or unfavorable outcome (death or LTX within 2 years following KPE (RLC)) using chart review and the biliary atresia and related diseases registry (www.bard-online.com (accessed on 1 January 2021)).

### 2.3. Redo-Kasai Portoenterostomy in One Infant

Follow-up data of one infant with redo-KPE were separately analyzed. KPE in this child was performed on day 42 of life, and on day 90 of life redo-KPE was performed due to the reoccurrence of jaundice. The samples retrieved at the second KPE were not included in group analyses, but intra-individually compared to the samples of the first KPE of this infant.

### 2.4. Sample Preparation and Multiplex Measurement

During the KPE, a small piece of periportal liver tissue was obtained, immediately snap-frozen in liquid nitrogen, and subsequently stored at −80 °C. Liver biopsies were achieved at the beginning of surgery, without the use of magnification, to reduce alterations due to liver trauma. Protein concentrations were measured (Pierce BCA Assay Kit, Thermo Scientific, Rockford, Illinois, USA) and adjusted to 500 µg/mL per sample. Biopsies were processed according to the protocol, previously reported in Egelkamp et al. [18]. Thirteen proteins functioning primarily as growth factors (BDNF, bFGF/FGF2, beta-NGF, CXCL12/SDF-1alpha, EGF, GM-CSF, IL-7, LIF, PDGF-bb, PIGF-1, SCF, VEGF-a, VEGF-d/FIGF) were assessed using ProcartaPlexTM Multiplex Immunoassays (Affymetrix, eBioscience) in accordance with manufacturer´s instructions [11]. In brief, the assay quantifies proteins based on the principle of sandwich ELISA with specific antibodies recognizing epitopes on a total of 13 different proteins. 

The growth factors were selected based on data in the literature. The role of FGF2, VEGF-a and -d, PDGF-bb, BDNF, beta-NGF, EGF, SCF, and Il-7 in the process of biliary atresia have been recently discussed in the literature. Furthermore, the additional growth factors (SD1-1alpha, LIF, GM-CSF, PIGF-1) were discussed to have a potential role in the process of liver fibrosis.

### 2.5. Data Analysis

Statistical analysis was performed using GraphPad Prism software version 8.0 (GraphPad Software, San Diego, USA) for calculation of Pearson´s correlation coefficient, and an unpaired *t*-test was used to analyze the survival with native liver (SNL) versus rapid liver cirrhosis (RLC) groups. Data are displayed as means and standard deviation (SD). *p* < 0.05 was considered statistically significant. An r_pears_ of >0.9 was considered a strong correlation.

A multidimensional principal component analysis (PCA) was performed, and individual growth factors were compared with the Qlucore Omics Explorer data analysis software (Qlucore, Lund, Sweden).

## 3. Results

### 3.1. Clinical Parameters in Infants with Biliary Atresia

A total of 22 infants (44.9%) were stratified into the group with a favorable outcome (jaundice-free survival with native liver (JF-SNL)), and 27 infants (55.1%) showed an unfavorable course of the disease (RLC) (Table 1). The mean age of the JF-SNL group at KPE was 61.8 (±22.7) days with mean total bilirubin levels at KPE of 174.9 µmol/L (±120.1 µmol/L). In the RLC group, the mean age at KPE was 69.4 (±28.0) days with a mean total bilirubin of 174.7 µmol/L (±96.1 µmol/L) at KPE. At the six month follow-up, mean bilirubin levels were 9.0 ± 6.2 µmol/L in patients with JF-SNL and 257.7 µmol/L (±203.6 µmol/L) in patients with RLC. Semiquantitative Ishak fibrosis scoring did not reveal significant differences at KPE in the SNL group (3.8 ± 1.6) compared to (4.1 ± 1.2) in infants with RLC (*p* = 0.28) [19].

Comparing the infants’ characteristics of the JF-SNL and RLC group, no statistical correlations were present for the age at KPE (*p* = 0.3) and the total bilirubin levels at KPE (*p* = 0.9), while the postoperative total bilirubin levels at six month follow-up in the JF-SNL was significantly lower in the JF-SNL group (*p* = 0.01).

### 3.2. The Protein Microenvironment of 13 Different Growth Factors in the Livers of BA Patients Does not Enable Differentiation of Clinical Outcome

**Figure 1:**
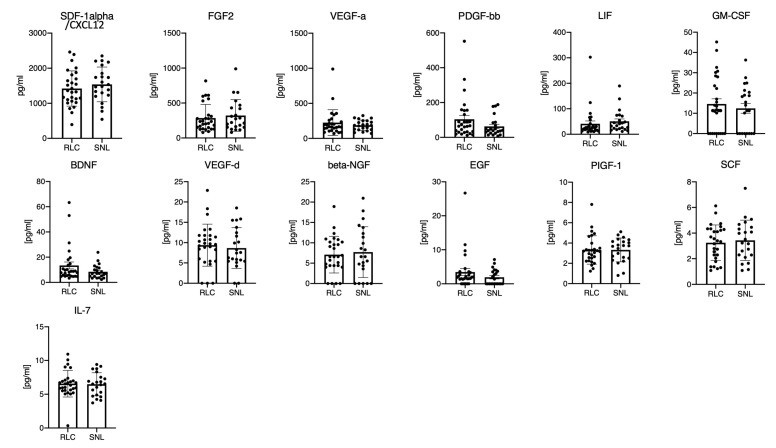
The protein microenvironment of 13 different growth factors in the livers of BA patients does not enable differentiation of clinical outcome. Legend: Multiplex measurement of 13 different growth factors from homogenized liver tissues of BA patients with favorable (SNL) or unfavorable course of disease (RLC). Each mark represents the value of an individual patient in the RLC or SNL group. The bar graph depicts the group mean; the error bar depicts the SD. Unpaired *t*-test yielded no statistically significant differences.

Thirteen mediators functioning predominately as growth factors were evaluated. High biomarker levels were measured for SDF-1alpha/CXCL12 (1473.0 ± 497.5 pg/mL), FGF2 (301.2 ± 207.8 pg/mL), and VEGF-a (209.0 ± 146.4 pg/mL). SDF-1alpha/CXCL12 (*p* = 0.41), FGF2 (*p* = 0.55), and VEGF-a (*p* = 0.37) did not show significant correlation with the later KPE outcome. The 10 further measured growth factor levels in the liver specimens also showed no significant differences concerning the patients’ postoperative liver disease progression (Figure 1, Table 2).

### 3.3. No Correlation of 13 Different Growth Factors in the Livers of BA Patients to the Pre- and Postoperative Total Bilirubin Levels, Ishak Score, or Age at KPE

None of the growth factor levels in the liver showed a significant correlation to clinical parameters at KPE or follow-up parameters (Table 3). In the case of the GF/chemokines with the highest hepatic levels, SDF-1alpha/CXCL12 did not correlate with the age at Kasai (r = 0.09), pre- (r = 0.02) and postoperative bilirubin (r = −0.12), or the Ishak score at KPE (r = 0.32). For FGF2 the correlation coefficients were unremarkable for age at KPE (r = 0.008), total pre- (r = 0.13) and postoperative bilirubin levels (r = 0.10), and the Ishak score at KPE (r = −0.099). In addition, no correlation was identified between VEGF-A and the age at KPE (r = −0.078), the pre- (r = 0.001) and postoperative bilirubin levels (r = 0.001), or the Ishak score (r = 0.17). High PDGF-bb levels were detected in the liver, but again did not correlate with the age at KPE (r = −0.04), the pre- (r = −0.15) and postoperative bilirubin levels (r = 0.08), or the Ishak score at KPE (r = −0.08). The same applied for all other growth factors in the liver.

### 3.4. The Protein Microenvironment of 13 Different Growth Factors in the Sera of BA Patients Does not Enable Differentiation of Clinical Outcome

**Figure 2:**
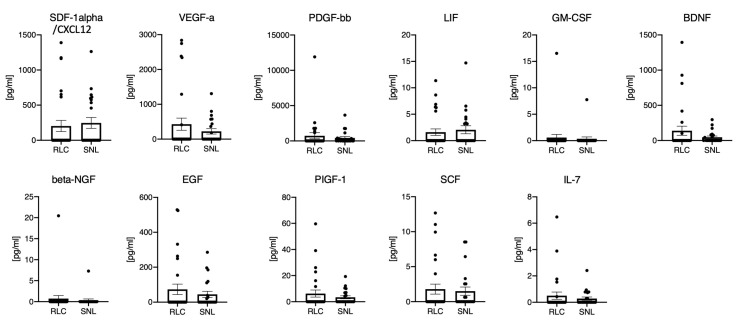
The protein microenvironment of 11 different growth factors in the sera of BA patients does not enable differentiation of clinical outcome. Legend: Multiplex measurement of 11 different growth factors from sera of BA patients with favorable (SNL) or unfavorable course of disease (RLC). For FGF2 and VEGF-D in most samples the levels were below the detection limits of the panel.

In comparison to the hepatic growth factor levels, the systemic response in the sera showed much higher inter-individual variations and were, in most cases, markedly lower in the serum compared to the liver (Figure 2, Table 4). In about half of the patients, serum values were below the detection limit of the employed assay. The highest levels were measured for PDGF-bb (604.8 ± 1813 pg/mL compared to 86.24 ± 96.97 pg/mL in the liver), VEGF-A (338.6 ± 737.8 pg/mL compared to 209.0 ± 146.4 pg/mL in the liver) and SDF-1alpha/CXCL12 (222.5 ± 394.0 pg/mL compared to 1473.0 ± 497.5 pg/mL in the liver). The assessed growth factor levels in serum of BA patients showed no significant differences concerning the patients’ postoperative liver disease progression (Figure 2, Table 4).

Each mark represents the value of an individual patient in the RLC or SNL group. The bar graph depicts the group mean, the error bar depicts the standard deviation. Unpaired *t*-test yielded no statistical significant differences.

### 3.5. Principle Component Analysis of 13 Different Growth Factors in the Livers of BA Patients and Statistical Analysis Regarding the KPE Outcome

**Figure 3:**
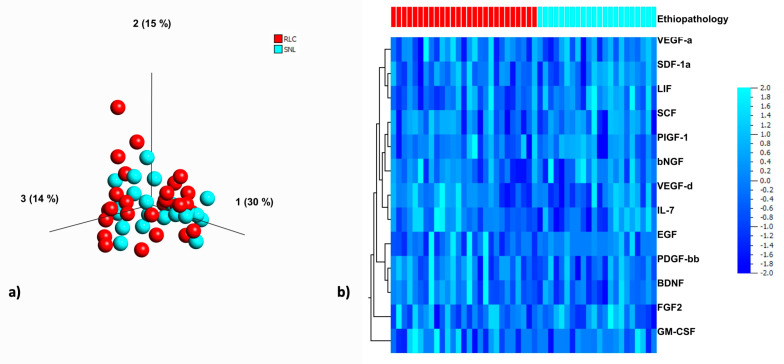
PCA of the growth factor microenvironment in livers of BA patients and clustered heat map for visualization and interpretation of the individual growth factor profile. Legend: (**a**) The scatterplot depicts a PCA of growth factors in BA patients with rapid liver failure (red) versus long term survival (blue). In this three-dimensional visualization, the blots distribute evenly without any grouping. Therefore, the variation in the expression of growth factors in the livers of BA patients at the time of KPE does not appear to be related to prognosis. (**b**) The heat-map visualizes the data with a grid matrix. The columns represent each patient/liver sample (red = rapid liver failure; blue = long term survival with native liver) and the rows represent each growth factor/chemokine. Intensity of the blue color visualizes changes of GF expression. No correlation was identified in the clustering.

Based on the patients’ outcome (SNL vs. RLC) no group or cluster could be identified by multidimensional PCA (Figure 3). In addition, the log transformed PCA data did not reveal significant correlations (Table 5).

### 3.6. Comparing the Hepatic and Systemic Growth Factors Levels in BA Patients at KPE

**Figure 4:**
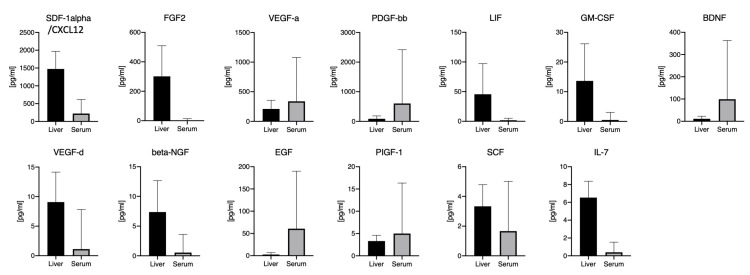
Comparing local (hepatic) and systemic (serum) levels in all 13 growth factors. Legend: Comparing multiplex measurements of 13 different growth factors from sera and livers of BA patients. The bar graph depicts the group means, the error bars depict the standard deviation.

None of the growth factors in the panel showed a strong correlation of the systemic (sera) and local (hepatic) levels. In detail, for SDF-1alpha/CXCL12 (r = 0.23), FGF2 (r= −0.12), VEGF-A (r = 0.02), PDGF-bb (r = 0.37), LIF (r = 0.0004), GM-CSF (r = 0.04), BDNF (r = 0.59), VEGF-D (r = 0.04), beta-NGF (r = 0.07), EGF (r = 0.34), PIGF-1 (r = 0.20), SCF (r = 0.22), and IL-7 (r = −0.08), the hepatic and systemic levels did not correlate (Figure 4).

### 3.7. Local and systemic growth factor milieu in a child undergoing redo-KPE

**Figure 5:**
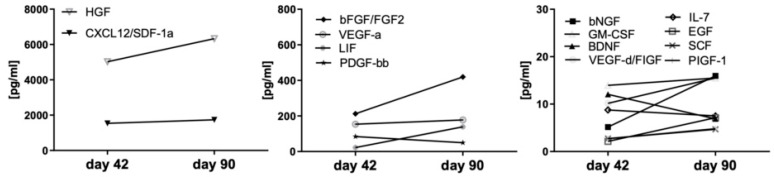
Stable pro-fibrotic growth factors in the liver of an infant undergoing Re-KPE. Legend: One infant underwent a first KPE at day 42 but had to undergo a Re-KPE at day 90. The protein microenvironment in the liver was assessed at both time points.

In one child following KPE at day 42 of life, jaundice reoccurred, necessitating re-KPE 48 days later. This single patient observation showed remarkedly stable expression levels of most growth factors over time despite deterioration of liver function. Only five growth factors showed some increase over time (bFGF (1.9-fold), VEGF-A (1.2 fold), LIF (6.3-fold), bNGF (3.1-fold), and EGF (3.4-fold)) (Figure 5). 

## 4. Discussion

Biliary atresia is the most common liver disease necessitating liver transplantation in childhood [2]. However, it is still unclear which infants have a long-term benefit from KPE and which show a rapid deterioration of liver function instead. Therefore, the prediction of disease course is desirable to enable timely referral to transplant centers.

Growth factors regulate cellular proliferation and differentiation via stimulating processes of cell division and survival. Various GF are known to play a significant role during regeneration processes following liver injury, e.g., by mediating liver stem cell differentiation. In cholestatic hepatopathies, pro- and anti-fibrotic signaling pathways are known to regulate hepatocyte and cholangiocyte differentiation. Growth factors mediating these pathways induce fiber–fiber, fiber–matrix, and matrix–matrix interactions, which possibly result in liver deterioration and end-stage liver cirrhosis [20,21,22]. Information on the growth factor milieus during the course of biliary atresia and their prognostic value for the disease outcome are scarce and have not yet been systemically assessed.

We therefore investigated the local (hepatic) and systemic (serum) growth factor levels in a cohort of 49 infants with biliary atresia. Levels were assessed during KPE and were correlated with the outcome after two years of follow-up, defined as either (jaundice-free) survival with native liver or liver transplantation.

The highest hepatic growth factor levels in our cohort were measured for stromal cell-derived factor 1alpha (SDF-1alpha/CXCL12), fibroblast growth factor 2 (FGF2), and vascular endothelial growth factor-a (VEGF-A) (Table 2). While increased serum SDF-1alpha and VEGF-A levels were present in some patients, the FGF2 levels in the serum were generally below the detection limit. SDF-1alpha/CXCL12 and its receptor CXCR4 stimulate the systematic movement of hematopoietic stem cells. It has been postulated that cells of hematopoietic origin are involved in liver regeneration during acute liver injury. These mechanisms were investigated in rat models exclusively; however, information on the role of SDF-1alpha/CXCL12 in human liver injury, fibrosis, and cirrhosis is missing [23]. High hepatic and lower serum SDF-1alpha/CXCL12 levels could indeed be explained by migration and the continuous cholestatic liver injury, but the missing increase of hepatic SDF-1alpha levels in the redo-KPE patient in between the procedures weakens this theory (Figure 5). FGF2 is a known contributor to liver fibrosis, and an anti-fibrotic effect has recently been hypothesized [24]. FGF serum expression during biliary atresia has been shown to be significantly higher in 65 BA patients compared to 12 healthy controls and showed the highest levels in jaundiced patients following KPE procedure [25]. Therefore, it has been concluded that the FGF expression could be associated with portal hypertension and the severity of liver damage [25]. However, no information on FGF levels within the liver tissue of infants with BA was available until now. Though the hepatic FGF2 expression showed higher values in the SNL outcome group, no statistical significance could be established (Figure 1). In addition, serum values showed no relation to the situation within the liver. In comparison to the previous studies, preoperative sonography in our cohort did not reveal portal hypertension in both outcome groups, being a potential reason for the low serum FGF levels at the time of Kasai procedure [25]. Furthermore, it is worth mentioning that we investigated the FGF2 levels at Kasai procedure, while studies reporting on high FGF levels in BA infants exclusively focus on the post-Kasai follow-ups [25].

For VEGF-A, hepatic profiling in biliary atresia has not shown an increased expression but been applied as a differentiation marker between biliary atresia and other cholestatic disorders in children [26,27]. In contrast, Fratta et al. measured lower hepatic VEGF-A values at KPE in 32 BA patients compared to 9 other cholestatic controls, concluding a BA-specific hypoxia–ischemia pathway [28]. Systemic VEGF is a non-specific trigger for both physiological and pathological angiogenesis, and overexpression during fibrotic and cirrhotic liver processes has already been detected in hepatopathies in childhood and in adults [29]. While recent analysis of FGF2 and VEGF-A levels were not correlated to the KPE outcome, our cohort did not show a significant correlation of the levels and the two year follow-up results (Figure 1 and Figure 2). Hepatic VEGF-D expression in our cohort was much lower than VEGF-A, and serum levels of VEGF-D were below the detection limits. VEGF-D is a known angiogenic factor in pediatric liver cancers, without any investigations concerning its expression in biliary atresia yet [30]. However, our hepatic and systemic VEGF-D values did not reveal any relevant relevance for assessing hepatic fibrosis, cirrhosis, or the prognosis for KPE outcome (Figure 1 and Figure 2).

Platelet-derived growth factor-BB (PDGF-bb) is an essential mediator during hepatitis-induced liver fibrosis and in alcoholic liver diseases. Serum PDGF-bb levels have been demonstrated to correlate with the degree of liver damage and fibrosis in adult patients [31]. In a rat model of cholestasis, PDGF had been shown to mediate chemotaxis of hepatic stellate cells towards bile duct structures, and thus facilitate a major contributor to liver fibrosis [32]. In our cohort, increased serum PDGF-bb levels were present, especially in the group with rapid liver failure and failed KPE procedure (Figure 2). However, PDGF-bb levels neither significantly correlated to the KPE outcome nor with the Ishak fibrosis levels in both outcome groups at KPE. Superina et al. and others have shown that semi-quantitative fibrosis grading at KPE is not correlated with postoperative outcome [11,14,33].

While the effects of leukemia inhibitory factor (LIF) during cholestatic disorders in humans have not been investigated yet, analysis in murine models showed a LIF involvement in liver stem cell differentiation [34]. Hepatic and systemic LIF levels in our cohort were lower compared to other GF and cytokines and did not correlate with KPE outcome (Figure 1 and Figure 2).

Brain-derived neurotrophic factor (BDNF) was recently used as a marker for the nutritional status in children and adolescents with liver cirrhosis due to biliary atresia [35]. The analysis in Wilasco et al. was limited to serum BDNF controls in 53 BA patients and 33 healthy controls, with correlation to the nutritional status and the height for age ration, but has indeed shown lower serum BDNF levels in the cirrhosis group. Decreased serum BDNF levels have also been detected in patients with hepatitis b-induced liver cirrhosis and were shown to correlate with the liver function [36]. These negative correlations of low BDNF serum levels and liver malfunction in infants and adults were not detectable in our cohort showing no significant differences of the hepatic and systemic BDNF levels comparing the SNL and RLC group (Figure 1 and Figure 2).

Beta nerve growth factor (b-NGF) is part of the biliary hyperplasia regulation during cholestasis and enhanced expression had been measured in primary sclerosing cholangitis [37]. While data on b-NGF in biliary atresia is not available in the current literature, in our cohort hepatic and systemic levels were low and did not correlate with the disease outcome (Figure 1 and Figure 2).

The epidermal growth factor (EGF) regulator pathway is a known contributor to hepatic fibrosis and cirrhosis, and serum EGF upregulation seemed to be associated with liver regeneration, reparation, and improved KPE outcomes in an analysis of 67 postoperative BA patients stratified for their outcome [38]. In our cohort, EGF liver and serum values were low and did present slightly higher levels in the BA patients with postoperative rapid liver cirrhosis.

Likewise, PIGF-1 and SCF hepatic and serum values did not show any significant differences for the KPE outcome. Placental derived growth factor 1 (PIGF-1) is a member of the VEGF family and pathological contributor to liver fibrosis and angiogenesis. Serum stem cell factor (SCF) was shown to be elevated in 57 BA patients compared to 30 healthy controls, and furthermore, seems to be correlated with the degree of liver injury and portal hypertension [39]. In our cohort of BA patients, the SCF levels were lower (in liver and sera) compared to other proteins in the panel and did not correlate with KPE outcome.

In addition, our cohort included one patient with redo-KPE (Figure 5). In this patient, KPE was performed at 42 days of life, and redo was done at 90 days of life. Remarkably the levels of the majority of growth factors between both procedures were stable, although cholestatic parameters and jaundice in this patient increased (Figure 5). Again, also in this patient, the levels of hepatic growth factors did not correlate with the liver function.

We also performed a principal component analysis to potentially identify clustering of the patients of the SNL and RLC cohorts. The three-dimensional visualization in the PCA did not present any grouping. The variation of growth factor expression in the livers of BA patients at the time of the KPE did, therefore, not appear to be related to the patients’ outcome.

Taken together, our study demonstrates high amounts of growth factors in livers from infants with BA at the time-point of KPE. No correlation with the outcome or serum values could be established. Thus, our data suggest that local or systemic growth factor levels at the time-point of KPE are unsuitable for prediction of the disease course and are a dismissible factor in disease progression.

## Figures and Tables

**Table 1:** Clinical parameters of the cohort.

Outcome	Rapid Liver Cirrhosis [RLC] (N = 27)	Survival with Native Liver [SNL] (N = 22)	*p*-Value
**Gender**			
Male	15	12	/
Female	12	10	/
Age at KPE (mean, in days)	69.4 ± 28.0	61.8 ± 22.7	0.3
Bilirubin levels at KPE (mean, in µmol/L)	174.7 ± 96.1	174.9 ± 120.1	0.9
Bilirubin levels at 6-month FU (mean, in µmol/L)	257.7 ± 203.6	9.0 ± 6.2	<0.01
ISHAK score at KPE [range 1–6]	4.1 ± 0.2	3.8 ± 1.6	0.3

**Table 2:** Local growth factor profile.

Growth Factors	Rapid Liver Cirrhosis (RLC) [pg/mL]	Survival with Native Liver (SNL) [pg/mL]	*p*-Value
SDF-1α/CXCL12	1422 ± 502.7	1539 ± 494.5	0.41
FGF2	285.4 ± 194.7	321.4 ± 226.3	0.55
VEGF-A	225.6 ± 184.3	187.9 ± 74.42	0.37
PDGF-bb	104.4 ± 117.4	63.11 ± 56.6	0.14
LIF	41.32 ± 57.37	50.93 ± 43.84	0.52
GM-CSF	14.59 ± 13.57	12.42 ± 11.17	0.55
BDNF	13.43 ± 14.29	8.43 ± 5.28	0.13
VEGF-D	9.38 ± 5.18	8.66 ± 5.03	0.62
Beta-NGF	7.09 ± 4.50	7.72 ± 6.23	0.68
EGF	3.47 ± 5.38	1.94 ± 2.29	0.22
PIGF-1	3.32 ± 1.41	3.32 ± 1.17	0.99
SCF	3.25 ± 1.38	3.44 ± 1.57	0.66
IL-7	6.56 ± 1.97	6.48 ± 1.71	0.87

**Table 3:** Correlating local growth factor levels with the age at KPE (days), preoperative bilirubin (µmol/L), postoperative bilirubin (µmol/L), and Ishak score.

Correlating GF with:	Age at KPE (r=)	Preoperative Bilirubin (r=)	Postoperative Bilirubin (r=)	ISHAK Grading (r=)
SDF-1α/CXCL12	0.09	0.02	−0.12	0.32
FGF2	0.008	0.13	0.10	−0.099
VEGF-A	−0.078	0.001	0.001	0.17
PDGF-bb	−0.04	−0.15	0.08	−0.08
LIF	−0.11	−0.03	−0.10	0.26
GM-CSF	0.36	−0.05	0.12	−0.03
BDNF	−0.05	−0.09	0.03	0.12
VEGF-D	0.06	0.17	0.18	0.08
Beta-NGF	0.16	−0.06	0.06	0.11
EGF	0.002	−0.11	0.11	0.13
PIGF-1	−0.09	−0.03	0.22	0.08
SCF	0.09	0.003	0.14	0.13
IL-7	0.27	0.28	0.08	−0.009

**Table 4:** Systemic growth factor profile.

Growth Factors/Chemokines	Rapid Liver Cirrhosis (RLC) [pg/mL]	Survival with Native Liver (SNL) [pg/mL]	*p*-Value
SDF-1α/CXCL12	203.6 ± 421.9	246.6 ± 363.7	0.71
FGF2	3.04 ± 16.1	*	*
VEGF-A	428.8 ± 931.6	223.8 ± 360.3	0.33
PDGF-bb	736.40 ± 2301.0	437.4 ± 901.8	0.57
LIF	1.61 ± 3.27	2.06 ± 3.59	0.64
GM-CSF	0.59 ± 3.12	0.35 ± 1.65	0.75
BDNF	139.8 ± 342.7	47.29 ± 82.9	0.22
VEGF-D	2.02 ± 8.89	*	*
Beta-NGF	0.73 ± 3.87	0.33 ± 1.55	0.65
EGF	73.27 ± 157.40	44.72 ± 81.48	0.44
PIGF-1	6.27 ± 14.38	3.40 ± 5.40	0.38
SCF	1.80 ± 3.79	1.51 ± 2.78	0.77
IL-7	0.50 ± 1.43	0.28 ± 0.57	0.49

* Below measuring limits of the panel.

**Table 5:** Log transformed data analysis of all growth factors/chemokines in liver tissue.

Growth Factors/Chemokine	*p*-Value	*q*-Value
SDF-1α/CXCL12	0.42385	0.86113
FGF2	0.56870	0.86113
VEGF-A	0.64716	0.86113
PDGF-bb	0.13977	0.60565
LIF	0.12720	0.60565
GM-CSF	0.79949	0.86113
BDNF	0.12117	0.60565
VEGF-D	0.33454	0.86113
Beta-NGF	0.33104	0.86113
EGF	0.49142	0.86113
PIGF-1	0.96485	0.96485
SCF	0.73271	0.86113
IL-7	0.73868	0.86113

## Abbreviations

BA	Biliary atresia
BARD	Biliary atresia and related diseases
BCA	Bicinchoninic acid assay
Beta-NGF	Beta-nerve growth factor
BDNF	Brain-derived neurotrophic factor
EGF	Epidermal growth factor
ELISA	Enzyme-linked immunosorbent assay
FGF2	Fibroblast growth factor 2
FIG	Figure
GM-CSF	Granulocyte-macrophage colony-stimulating factor
IL	Interleukin
KPE	Kasai portoenterostomy
LIF	Leukemia inhibitory factor
LTX	Liver transplantation
MP	Medical products
NCBI	National Center for Biotechnology Information
PCA	Principle component analysis
PDGF-bb	Platelet-derived growth factor receptor
PIGF-1	Placenta growth factor 1
RLC	Rapid liver cirrhosis
SCF	Stem cell factor, Kit-ligand
SD	Standard deviation
SDF-1alpha/CXCL12	Stromal cell-derived factor 1, CXCL12
SNL	Survival with native liver
TNF	Tumor necrosis factor
VEGF-A,-D	Vascular endothelial growth factor a, -d
